# The development of a guide to borderline orthodontic need

**DOI:** 10.1186/s40510-022-00407-6

**Published:** 2022-04-18

**Authors:** Ariane Sampson, Huw G. Jeremiah, Nicholas N. Lai, Robert Kirschen

**Affiliations:** 1 Department of Orthodontics, Clinic 8, Addenbrookes Hospital, Cambridge University Hospital NHS Foundation Trust, Hills Road, Cambridge, CB2 0QQ UK; 2 The Diamond Smile Studio, 182 High St. Harlesden, London, NW10 4ST UK; 3 Department of Orthodontics, Institute of Dentistry, Barts and The London School of Medicine and Dentistry, Queen Mary University of London, Whitechapel, London, E1 2AD UK

**Keywords:** IOTN, Aesthetics, Index, Borderline, Malocclusion, Orthodontic treatment need

## Abstract

**Objective:**

To produce a guide for dentists and orthodontists to determine orthodontic treatment need in borderline cases (dental health component DHC 3) and to compare views of different panels of judges on treatment need.

**Materials and methods:**

Prospective, observational study. Photographs of one hundred subjects displaying borderline occlusal traits (DHC3) were collected. Three panels of judges consisting of 25 orthodontists, 25 dentists and 25 lay persons assessed photographs based on orthodontic treatment need on aesthetic grounds.

**Results:**

Spearman’s correlation coefficient showed no statistical difference between the panels of judges (*p* < 0.001). The judges identified a ‘high need’ for treatment on aesthetic grounds for those with anterior open bites and reverse overjets. Kappa analysis showed moderate intra-rater agreement for the orthodontic and dental panels of judges (*k* = 0.47 and 0.45, respectively) and fair agreement (*k* = 0.26) for the lay panel, highlighting the intrinsic difficulty of assessing borderline malocclusions.

**Conclusion:**

There was no statistical difference in the way the orthodontic, dental and lay panels of judges perceived treatment need for DHC 3 cases. Anterior open bites and reverse overjets were predominantly found to be in high need of treatment by all panels of judges. A ‘Guide to Borderline Orthodontic Need’ (GBON) is proposed consisting of 8 photographs of subjects with borderline occlusal traits (DHC3) determined unambiguously by lay, dental and orthodontic panels as either ‘needing’ or ‘not needing’ orthodontic treatment on aesthetic grounds. It is anticipated that this will assist users to make judgments on aesthetic grounds on the need for treatment in borderline cases.

## Introduction

Orthodontic treatment has been available in the UK within the National Health Service (NHS) since its foundation in 1948. The availability of orthodontic treatment for children at no cost to the parent/guardian has been a powerful factor in generating demand. However, in publicly funded health care systems, resources are frequently insufficient to meet this demand and some form of regulation is often employed, for example a resource allocation system based on prioritising malocclusion, as in the UK.

Following the Schanschieff Report in 1986 [1], the Index of Orthodontic Treatment Need (IOTN) was developed to assess the need for treatment. The IOTN, based on the Index of Treatment Priority used by the Swedish Dental Board [2], consists of the dental health component (DHC) and the aesthetic component (AC) [3]. Both components help to identify individuals who are most in need of or would most benefit from orthodontic treatment, and the Index continues to be used worldwide [4]. It has been widely accepted that malocclusions with DHC scores of 1 and 2 do not require treatment and that those with scores of 4 and 5 have a definite need for treatment. In between, malocclusions with a score of 3 are regarded as moderate or borderline [5]. Whilst the reproducibility of the DHC, based on the significance of various occlusal traits, has been shown to be good, there is often poor agreement for the AC [3, 6, 7], a chart of ten photographs ranging (1 to 10) from the most to the least aesthetic.

Treatment decisions for borderline malocclusions, including on whether to treat [8–10], are among the most difficult. Whilst the IOTN can categorise treatment need into broad groups, it is difficult to use the AC to discern which borderline cases have a justifiable need for treatment. Most significantly, the AC of the IOTN was not created or validated with the specific intent that photographs 5 and 6 would determine whether patients would gain access to NHS treatment or not. More specific guidance is appropriate for borderline cases.

The aim of this study was to create a guide, supported by lay and professional opinion, to help identify the need for orthodontic treatment in borderline cases.

## Materials and methods

### Ethical approval and consent

Ethical approval was obtained from the East London and the City Local Research Ethics Committee prior to starting the study. Eligible participants were provided with verbal and written information, and signed consent was obtained prior to participation.

### Sample selection

Statistical input advised that a sample size of 100 subjects to be assessed would be sufficient to provide a variety of traits from within the narrow spectrum of borderline malocclusions. Subjects were recruited from ‘New Patient’ Orthodontic Consultant Clinics held at the Royal London Hospital and examined by the chief investigator (NL). The following inclusion criteria were met:

- A score of 3 in the dental health component of the IOTN:
  - Increased overjet greater than 3.5 mm but less than or equal to 6 mm, with incompetent lips (3a)
  - Reverse overjet greater than 1 mm but less than or equal to 3.5 mm (3b)
  - Anterior or posterior crossbites with greater than 1 mm but less than or equal to 2 mm discrepancy between retruded contact position and intercuspal position (3c)
  - Contact point displacements greater than 2 mm but less than 4 mm (3d)
  - Lateral or anterior open bite greater than 2 mm but less than or equal to 4 mm (3e)
  - Deep overbite complete on gingival or palatal tissues but no trauma (3f)
- Absence of or only mild gingival pigmentation
- Permanent dentition or in the late mixed dentition with only retained second deciduous molars
- Unrestored or minimally restored dentition

### Photographs

The chief investigator (NL) attended a calibration course on the use of Occlusal Indices, which included the IOTN. Each participant’s DHC of the IOTN was recorded, and a single frontal intra-oral photograph and a set of study model impressions were taken.

Once the sample of 100 photographs had been gathered, they were rotated and cropped as necessary to ensure horizontal occlusal planes to make them consistent with each other. The photographs were set up in random order on a Microsoft Power Point slide show and clearly numbered so they could be easily identified and scored by individual judges. Each time the photographs were shown, the sequence was re-randomised to reduce the order effect.

A pilot study involving ten orthodontists, dentists and adult patients was undertaken to help determine the best layout for the questionnaire for the study. The questionnaire they filled out determined that judges preferred to view each photograph for 10 s to allow full appreciation of the photograph without it taking too long. They also provided feedback on the layout of the questionnaire to be used in the study.

### Error study

An error study was incorporated into the main study by repeating every fifth photograph at the end of the slide show, thus increasing the total number of photographs from 100 to 120. The judges were unaware that their reproducibility was under scrutiny.

### Questionnaire

A questionnaire was created to determine whether, in the judge’s opinion, the malocclusions shown in each of the 120 photographs warranted treatment for reasons of ‘appearance’, with four tick-box options: definitely no; borderline no; borderline yes; definitely yes.

At no time were the judges given any information about the borderline nature of the malocclusions to be assessed or about occlusal traits.

### Judges

Three panels of 25 judges were recruited:

- The orthodontic panel (specialist orthodontic registrars, specialist orthodontists and consultant orthodontists from the orthodontic departments at The Royal London Hospital, Essex County Hospital, Guy’s Hospital and King’s College Hospital)
- The dental panel (qualified dentists with no postgraduate orthodontic training or experience, including periodontists, prosthodontists, restorative consultants, implantologists and dentists with specialist interests from the paedodontic, restorative, oral and maxillofacial surgery, and oral medicine departments at The Royal London Hospital and Guy’s Hospital)
- The lay group (any person who had not undergone any previous orthodontic treatment, whose children were not undergoing orthodontic treatment, whose occupation was not in the dental field and had no extensive dental knowledge, recruited from hospital outpatient departments, secretaries and radiologists at the Essex County Hospital).

Judges for each panel were selected by the chief investigator and identical instructions were given individually prior to grading the photographs in the slide show. Special precautions were taken not to inform the orthodontic and dental judges of the objective features or borderline nature of the photographs to avoid bias. The chosen independent judges were not involved with the study and had no prior knowledge of the study. They were chosen based on their relevant specialty (for the orthodontic and dental panels) and their non-involvement with the study. Once the slide show had started, assessments were carried out in silence and in a single sitting. The screen size, brightness and contrast were kept identical throughout to ensure the same conditions.

### Statistical analysis

Data from the main study and scores from the error study were analysed by the Statistical Package for Social Sciences computer software (SPSS Inc., Michigan Avenue, Chicago, IL, USA), using means, Spearman’s correlation analysis, and kappa analysis. A statistician was consulted regarding study design, statistical analysis and interpretation of the results.

## Results

### Correlations between panels

Spearman’s bivariate correlation coefficients (Table 1) were used to demonstrate a statistically significant positive correlation in the way the three panels of judges graded the photographs and treatment need (*p* < 0.001), showing inter-group agreement and enabling the joint ranking of the photographs by merging the three panels of judges.

**Table 1:** Spearman’s bivariate correlation coefficients comparing scores of three panels of judges

	Spearman’s correlation coefficient	Statistical significance (2-tailed)
Orthodontic panel versus dental panel	0.961	*p* < 0.001
Dental panel versus lay panel	0.838	*p* < 0.001
Orthodontic panel versus lay panel	0.865	*p* < 0.001

### Mean scores

The means for each photograph were calculated for the panels of 25 orthodontic judges, 25 dental judges and 25 lay judges and then ranked according to their mean scores. The more negative the value, the lower the need for treatment. The more positive the value, the greater the need for treatment.

The three panels of judges were merged to produce a single ranking (Table 2). The treatment need of each individual photograph was determined on the grounds of aesthetics. From information on occlusal traits, it is possible to analyse whether certain traits are likely to justify treatment on aesthetic grounds. Six out of the seven highest ranked photographs deemed as ‘definitely in need of treatment’ displayed occlusal traits of reverse overjets (highlighted in green) or anterior open bites (highlighted in yellow). Thirty-two of the first 33 photographs that were deemed as ‘least in need of treatment’ displayed occlusal traits of increased overjets and contact point displacements.

**Table 2:**
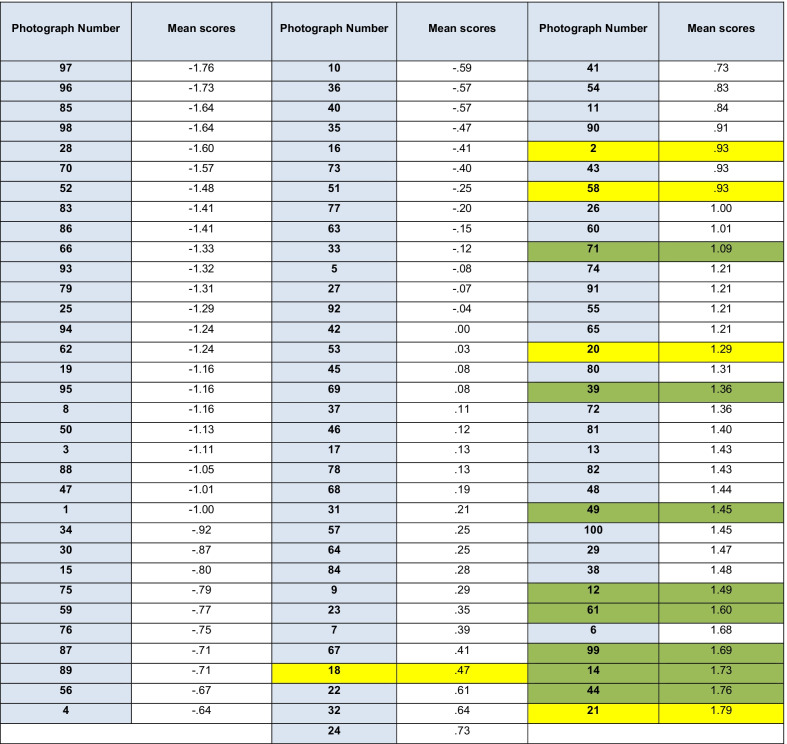
Ranked means of 100 photographs as assessed by the merged panels of 75 judges (green highlight = reverse overjet and yellow highlight = anterior open bite)

### Intra-rater agreement

In addition to the original 100 borderline photographs displaying DHC 3 malocclusions, 20 repeat pictures were added to the end of the slide show as part of the error study. Kappa analysis showed that the orthodontic and dental panels had moderate intra-rater agreement. The lay group demonstrated fair intra-rater agreement (Table 3).

**Table 3:** Percentages of perfect intra-rater agreement and kappa scores for the panels of judges

Panels	Perfect agreement (%)	kappa score
Orthodontic	61	0.47
Dental	59	0.45
Lay	46	0.26

## Discussion

### Panels of judges

Previous studies have shown a discrepancy between lay and professional judgement of dental aesthetics and need for treatment, with professionals rating dental appearance more critically [6, 10]. This study showed a statistically significant positive correlation in the way orthodontists, dentists and lay people graded the photographs and assessed treatment need.

### Inter-rater agreement

To achieve validation, we examined inter-rater and intra-rater agreement. Spearman’s bivariate correlation coefficients used to measure the inter-rater agreement showed statistically significant agreement between all three panels of judges.

### Intra-rater agreement

The kappa test for the orthodontic and dental panel of judges showed moderate reliability and the lay panel showed fair reliability. This is in keeping with the intrinsic difficulty of assessing borderline cases, as found in previous studies [8], particularly for lay people who have no familiarity with the critical assessment of dental aesthetics.

### Guide to borderline orthodontic need (GBON)

**Fig. 1:**
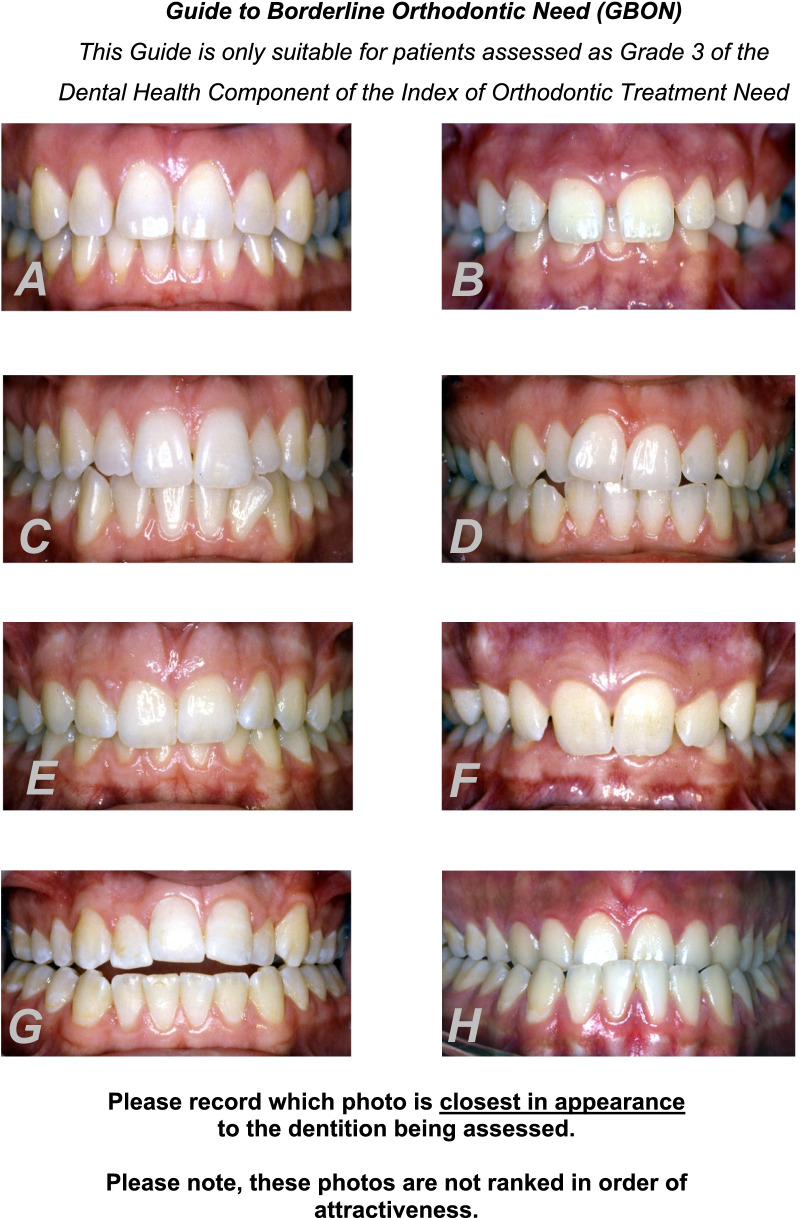
Guide to borderline orthodontic need (GBON)

This study identified occlusal traits in borderline malocclusions deemed worthy of orthodontic treatment on aesthetic grounds, as determined and agreed by orthodontists, dentists and lay people alike. Anterior open bites and reverse overjets were ranked highly in need of treatment on aesthetic grounds by all three panels. This information justifies the creation of a proposed Guide to Borderline Orthodontic Need (GBON), an index devised and validated specifically for use on patients scoring 3 in the DHC of the IOTN (Fig. 1). It is not an appropriate guide for patients with other DHC scores. The GBON does not aim to ‘grade’ dental aesthetics but includes a range of dental appearances that lay, dental and orthodontic panels agreed either ‘need’ or ‘do not need’ orthodontic treatment on aesthetic grounds. It consists of eight photographs: three photographs (A, C and E) are typical of occlusions that do not require treatment on aesthetic grounds, and five photographs (B, D, F, G and H) represent occlusions that could benefit from treatment on aesthetic grounds.

The deliberate inclusion of various occlusal traits in the proposed guide runs counter to the basic principle of aesthetics grading found in the AC of the IOTN. It is important to note that the presentation of the GBON series of eight photographs makes no mention of occlusal traits, enabling the user to make judgments on aesthetic grounds for, or with, patients and parents on the need for treatment.

A further difference from the AC of the IOTN is that the eight photographs are not a scale of attractiveness. Users are required to assess which photograph is “closest in appearance” to the borderline malocclusion being assessed.

This proposed guide was created through a structured and methodical process that has identified various borderline cases that would benefit from treatment on aesthetic grounds. The clinical application of the GBON has been evaluated in a separate prospective investigation involving the assessment of borderline malocclusions. This investigation also compared the performance of the GBON with the AC of the IOTN.

## Limitations

Assessing dental attractiveness is subjective and may be systematically related to the judge’s demographic background. It has been suggested that lay people often rank photographs for dental and facial attractiveness according to their own demographic background [11]. In future, the role of age, ethnicity and gender in the assessment of dental aesthetics should be investigated.

There were very few subjects with spaced dentitions, an occlusal trait deemed unfavourable particularly in Caucasian populations [12–15], and not represented in the AC of the IOTN [16]. The aesthetics of spacing should be researched further.

There are intrinsic weaknesses in the assessment of dental aesthetics by means of a static anterior view, particularly for subjects with well aligned teeth but increased overjets (for example, photograph A). Smile aesthetics involves more than just dental aesthetics and should be assessed by a series of photographs from various angles without cheek retractors or, better still, by moving video images.

## Conclusions

There was no statistical difference in the way the orthodontists, dentists and lay people perceived treatment need on aesthetic grounds for DHC 3 cases.

Anterior open bites and reverse overjets were found to be in high need of orthodontic treatment by all panels of judges.

The ‘Guide to Borderline Orthodontic Need’ (GBON) is proposed based on photographs of varying dental appearances, both needing and not needing treatment. It can help clinicians and patients identify the need for orthodontic treatment specifically in borderline cases.

## Data Availability

The datasets generated and analysed during the current study are not publicly available due them being collected on paper but may be available from the corresponding author on reasonable request.
